# Regulatory roles of the LysR-type transcriptional regulator GltC in metabolism, stress tolerance, and virulence of *Listeria monocytogenes*

**DOI:** 10.1128/spectrum.00391-26

**Published:** 2026-08-21

**Authors:** Seto C. Ogunleye, Monzur Chowdhury, Hossam Abdelhamed

**Affiliations:** 1 Department of Comparative Biomedical Sciences, Mississippi State University 542196, Mississippi State, Mississippi, USA; University of Dundee, Dundee, United Kingdom

**Keywords:** *Listeria monocytogenes*, metabolic regulation, nitrogen assimilation, oxidative stress tolerance, virulence

## Abstract

*Listeria monocytogenes* is a gram-positive facultative intracellular bacterium that causes listeriosis in humans and animals. Survival in diverse environments and host-associated niches requires coordinated regulation of metabolism, stress responses, and virulence*.* The LysR-type transcriptional regulator GltC is predicted to regulate glutamate metabolism, yet its broader contribution to metabolic regulation and pathogenicity in *L. monocytogenes* remains unclear. Here, we explored the role of GltC in nitrogen utilization, central carbon metabolism, stress tolerance, and virulence. Deletion of *gltC* had no detectable effect on growth under nutrient-rich, nitrogen-defined conditions or at acidic and alkaline pH, indicating that GltC is not required for basal fitness. Instead, loss of *gltC* triggered extensive transcriptional remodeling of nitrogen assimilation pathways, including altered expression of genes involved in glutamate synthesis, ammonium transport, and ethanolamine utilization, as well as condition-dependent changes in tricarboxylic acid cycle-associated genes. The Δ*gltC* mutant exhibited enhanced tolerance to oxidative stress, with improved growth and survival during hydrogen peroxide challenge and increased expression of oxidative stress defense and DNA repair genes. Deletion of *gltC* led to broad upregulation of the PrfA virulence regulon and a modest increase in hemolytic activity. The Δ*gltC* strain displayed significantly higher bacterial burdens in the spleen and liver in a murine infection model. Collectively, these results suggest that GltC contributes to the coordination of nitrogen metabolism and oxidative stress adaptation, while influencing bacterial fitness during systemic infection.

**IMPORTANCE:**

Pathogenic bacteria must constantly balance nutrient acquisition, metabolic activity, and virulence to persist in changing environments. *Listeria monocytogenes* causes severe foodborne disease and must adapt to changing nutrient and stress conditions during infection. Understanding how metabolism influences virulence is critical for identifying new strategies to limit bacterial survival in the host. This study shows that GltC contributes to the coordination of nitrogen metabolism, oxidative stress adaptation, and bacterial fitness during *L. monocytogenes* infection. Although GltC is not required for basic growth, its loss alters nitrogen metabolic pathways, enhances oxidative stress tolerance, and is associated with increased bacterial burdens during murine infection. These findings provide new insights into how metabolic regulation influences bacterial adaptation and fitness during host infection.

## INTRODUCTION

*Listeria monocytogenes* is a major foodborne pathogen of humans and animals and is distinguished by its ability to survive, persist, and adapt across diverse environmental niches (1). Infection with *L. monocytogenes,* termed listeriosis, can manifest as self-limiting gastroenteritis in healthy individuals but often progresses to septicemia, abortion, and severe neurological disease in immunocompromised populations, pregnant women, and neonates (2, 3). The capacity of *L. monocytogenes* to switch between saprophytic and pathogenic lifestyles is governed by highly coordinated regulatory networks that integrate environmental cues, metabolic status, and host-derived signals (4).

Among the regulatory systems that contribute to this adaptability are LysR-type transcriptional regulators (LTTRs), which contribute to cellular metabolism, virulence regulation, cell envelope homeostasis, flagellar function, motility, and stress responses in *L. monocytogenes* (5–8). LTTRs represent one of the largest families of DNA-binding proteins in prokaryotes, with more than 800 members identified based on their amino acid sequences (8, 9). In *L. monocytogenes,* LTTRs function as activators or repressors depending on ligand availability and environmental context, enabling dynamic transcriptional control in response to metabolic cues (8, 10–12).

One of the few LTTRs that has been functionally characterized in *L. monocytogenes* is GltC, a transcriptional regulator primarily associated with glutamine and glutamate metabolism (10, 12). Structurally, GltC contains conserved HTH_1 and HTH_5 DNA-binding motifs within its N-terminal domain (amino acids 1–55), while the C-terminal domain mediates ligand binding to a wide range of effector molecules, including amino acids, sugar phosphates, organic acids, metal cations, NADPH, ATP, citrate, fatty acyl-CoA, reactive oxygen species, and second messengers such as c-di-GMP and (p)ppGpp (13, 14). This structural versatility positions GltC as a potential metabolic sensor capable of integrating nutrient and stress signals.

**Fig 1:**
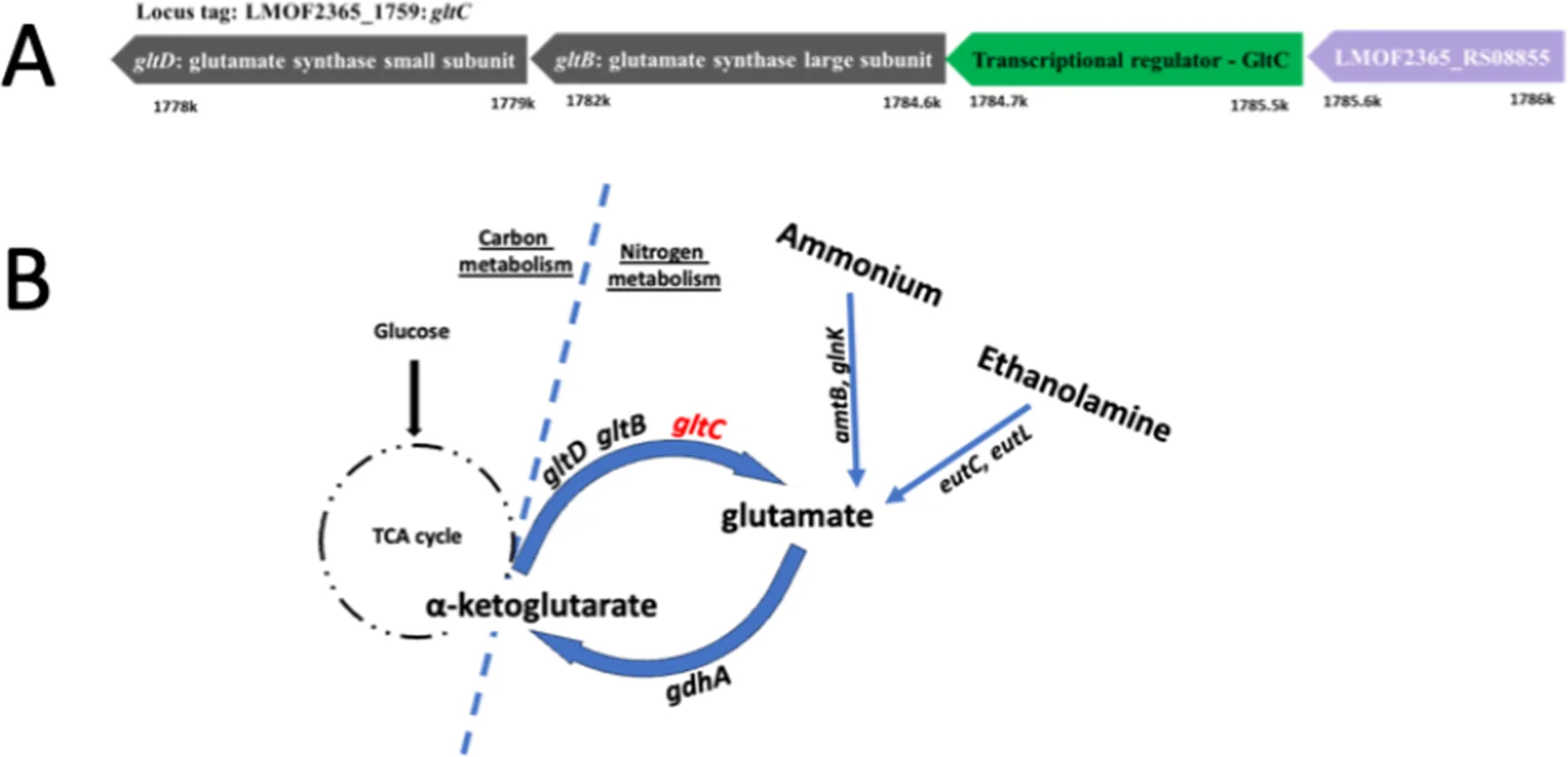
Organization of the *gltDBC* locus and its role in glutamate and α-ketoglutarate metabolism in *L. monocytogenes*. (**A**) Schematic representation of the genomic organization of the *gltDBC* genes, highlighting the position of *gltC* relative to *gltB* and *gltD*, which encode the large and small subunits of glutamate synthase, respectively. (**B**) Proposed model illustrating the role of the *gltDBC* system in regulating glutamate synthesis through control of the interconversion between α-ketoglutarate and glutamate. The model also depicts the integration of alternative nitrogen sources, including ammonium and ethanolamine, into glutamate-centered metabolism under GltC-dependent regulation. Solid arrows indicate the direction of metabolic flux between α-ketoglutarate and glutamate, whereas dashed lines represent putative regulatory interactions mediated by GltC.

The *gltC* gene is located immediately upstream of the *gltB* and *gltD* genes, which encode the large (*gltB*) and small (*gltD*) subunits of glutamate synthase, an essential enzyme complex required for glutamate biosynthesis (12). In *L. monocytogenes,* glutamate metabolism plays a critical compensatory role by generating α-ketoglutarate, thereby bypassing the absence of α-ketoglutarate dehydrogenase in the tricarboxylic acid (TCA) cycle. This metabolic adaptation maintains TCA cycle flux and supports the production of metabolic intermediates required for energy generation and biosynthetic processes (Fig. 1) (15–18).

Glutamate synthase (GOGAT; GltB/GltD) is a central enzyme complex that links nitrogen assimilation to carbon metabolism by catalyzing the conversion of α-ketoglutarate and glutamine into glutamate. Expression of *gltBD* is tightly regulated by cellular carbon and nitrogen availability, thereby integrating signals from preferred carbon sources and nitrogen donors such as glutamine and ammonium, as well as alternative nitrogen sources under specific metabolic conditions (19). Previous studies have shown that under glutamine-rich conditions, transcription of the *gltBD* operon is strongly activated by GltC activation, whereas glutamine limitation suppresses this regulatory pathway (20). In parallel, the global nitrogen regulator GlnR plays a central role in maintaining nitrogen homeostasis by repressing multiple genes involved in nitrogen uptake and assimilation during nitrogen-replete conditions, including the *glnRA* operon, glutamate dehydrogenase (*gdhA*), the ammonium transport system (*amtB*), and the regulatory protein GlnK (*glnK*). Through this repression, GlnR limits excess nitrogen assimilation when preferred nitrogen sources are abundant, thereby preventing unnecessary metabolic expenditure and maintaining intracellular nitrogen balance (12, 19, 21).

Beyond its role in central metabolism, glutamate metabolism is a key determinant of multiple physiological and pathogenic traits in *L. monocytogenes*, including biofilm formation, oxidative stress resistance, intracellular survival, and virulence. A critical connection between metabolic status and virulence regulation is mediated by the transcriptional regulator CodY, whose DNA-binding activity is governed by intracellular branched-chain amino acid and GTP availability (22, 23). Under nutrient-limited conditions, reduced CodY activity relieves repression of virulence-associated pathways, thereby permitting activation of Positive Regulatory Factor A (PrfA), the master regulator of virulence gene expression in *L. monocytogenes*. PrfA governs a coordinated virulence program encoded largely within *Listeria* Pathogenicity Island 1, including *prfA*, the phosphatidylinositol-specific phospholipase C (*plcA*), the cholesterol-dependent cytolysin listeriolysin O (*hly*), the zinc metalloproteinase Mpl (*mpl*), the actin assembly-inducing protein ActA (*actA*), the phosphatidylcholine phospholipase C (*plcB*), and the internalins InlA (*inlA*) and InlB (*inlB*) (24–26). Collectively, these PrfA-regulated factors are essential for host cell invasion, intracellular replication, cell-to-cell spread, and systemic infection (27–29).

Although LTTRs have been broadly implicated in the regulation of virulence, biofilm formation, and stress responses in *L. monocytogenes*, specific interplay between individual LTTRs, such as GltC, and the PrfA regulatory network remains poorly understood. In particular, how metabolic regulators that govern glutamate–glutamine homeostasis intersect with PrfA-dependent virulence pathways has not been fully elucidated. We hypothesized that GltC plays a significant role in coordinating metabolic state with virulence and stress adaptation in *L. monocytogenes*. The primary objective of this study was to define the contribution of GltC to nutrient utilization, biofilm formation, oxidative stress response, and virulence. Specifically, we sought to determine how loss of GltC impacts the transcriptional regulation of key metabolic genes involved in virulence regulation, glutamine, ammonium and ethanolamine metabolism, the TCA cycle, and oxidative stress defense. We further evaluated how these transcriptional changes affect metabolic adaptation, stress tolerance, and virulence-associated phenotypes. Through this integrated approach, we aimed to clarify the role of GltC in shaping the physiological and pathogenic adaptability of *L. monocytogenes*.

## RESULTS

### Deletion of *gltC* does not impair fitness of *L. monocytogenes* under nutrient-rich, nitrogen-defined, or pH-stress conditions

**Fig 2:**
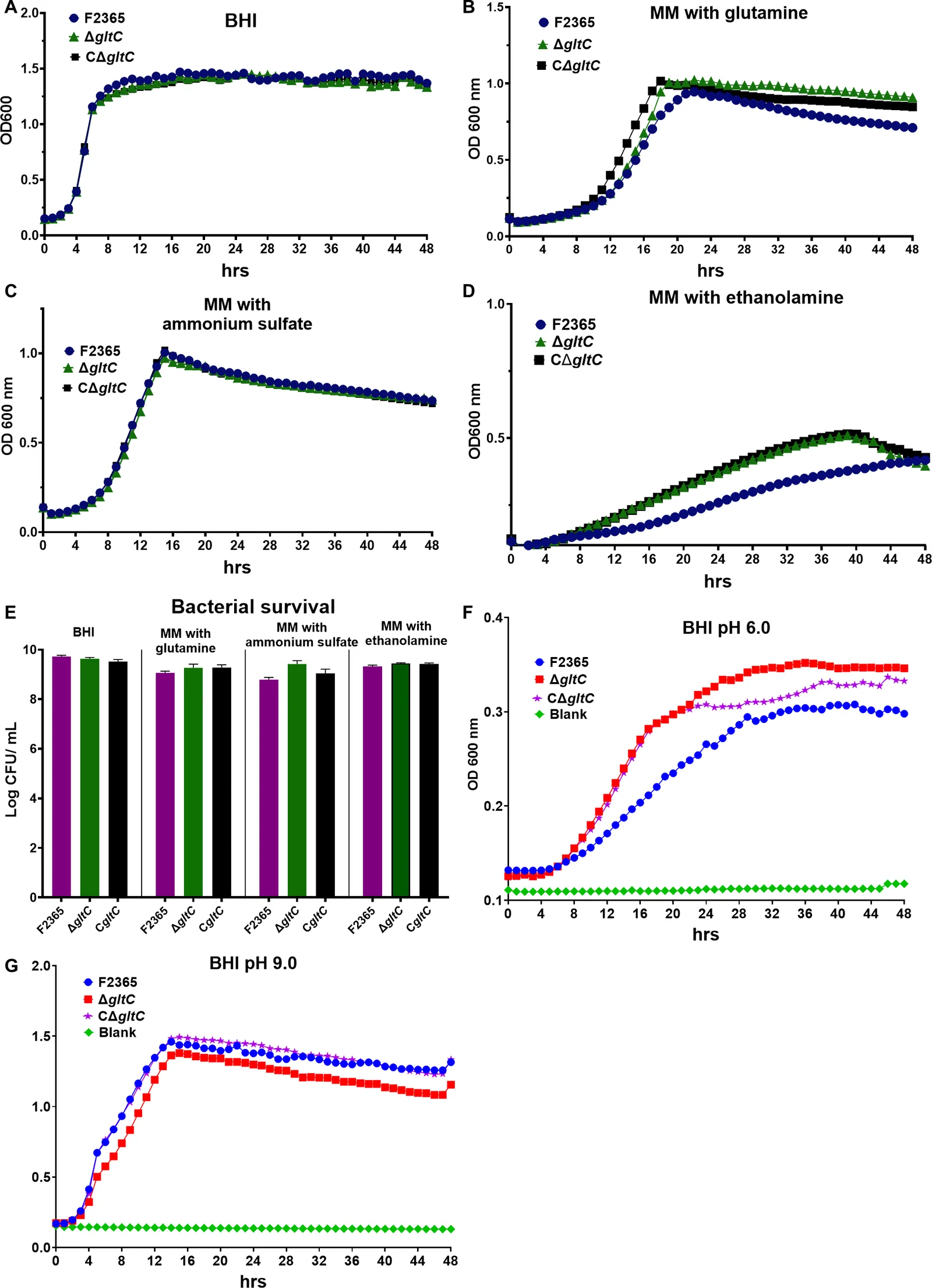
Comparison of growth kinetics and bacterial fitness of the Δ*gltC* relative to the wild-type F2365 strain under nutrient-rich, nitrogen-defined, and pH-stress conditions. Growth curves were assessed in BHI broth (**A**), MM supplemented with L-glutamine (**B**), ammonium sulfate (**C**), or ethanolamine (**D**), with corresponding viable bacterial loads (log_10_ CFU/mL) quantified after 18–24 h (**E**). Growth was also assessed in BHI adjusted to pH 6.0 (**F**) or pH 9.0 (**G**). Data represent mean ± SEM from three independent biological replicates, each comprising four technical replicates per condition. Statistical significance was determined using Student’s *t*-test comparing Δ*gltC* with the wild type. No significant differences were observed under any condition, indicating that deletion of *gltC* does not impair basal growth fitness or tolerance to acidic and alkaline pH.

The Δ*gltC* strain exhibited growth kinetics comparable to wild-type F2365 when cultured in nutrient-rich brain heart infusion (BHI) broth, indicating that *gltC* is not essential under these conditions (Fig. 2A). Similarly, no significant differences in growth profiles, as measured by optical density at 600 nm (OD_600_), were observed between the Δ*gltC* strain and wild-type F2365 when grown in minimal medium (MM) supplemented with glutamine (20 mM; Fig. 2B), ammonium (30 mM; Fig. 2C), or ethanolamine (30 mM; Fig. 2D) as sole nitrogen sources. Viable bacterial counts determined 18–24 h after plating confirmed comparable bacterial loads, with the Δ*gltC* mutant showing only marginal increases of 0.1, 0.1, 0.4, and 0.2 log_10_ colony-forming units (CFUs) in BHI, glutamine, ammonium, and ethanolamine compared to wild-type F2365, respectively (Fig. 2E). These differences were not statistically significant (*P* > 0.05).

To further assess whether *gltC* contributes to fitness under environmental stress, growth of Δ*gltC* and wild-type F2365 strains was assessed in BHI adjusted to acidic (pH 6.0) or alkaline (pH 9.0) conditions. Growth of the Δ*gltC* and wild-type strains was similarly impaired at pH 6.0, with no significant differences observed between the two strains (Fig. 2F). Likewise, growth of the Δ*gltC* strain at pH 9.0 was comparable to that of the wild-type strain (Fig. 2G). These results indicate that *gltC* deletion does not compromise tolerance to either acidic or alkaline conditions.

### Deletion of *gltC* induces widespread transcriptional remodeling of nitrogen and central carbon metabolism

**Fig 3:**
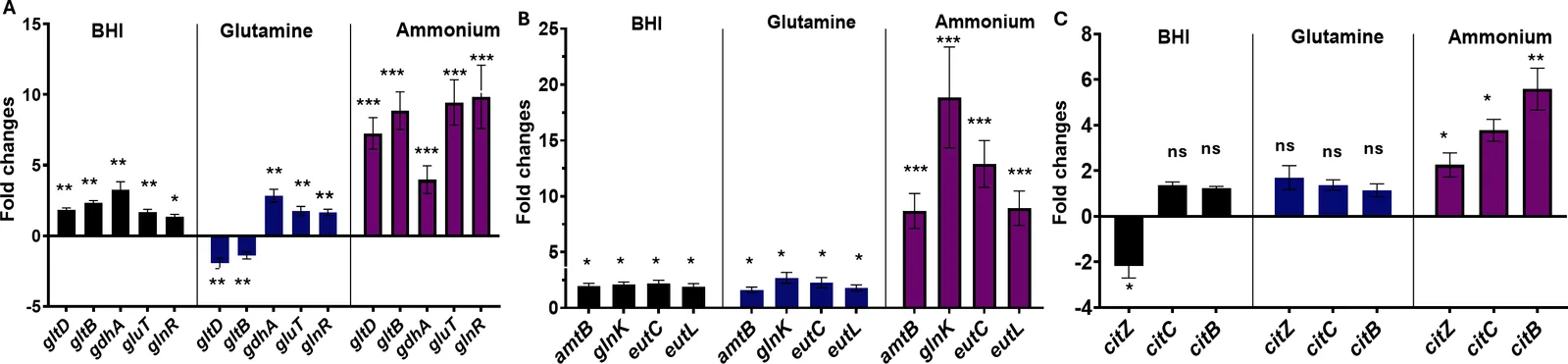
Transcriptional profiling of nitrogen metabolism and central carbon metabolism in the Δ*gltC* relative to wild-type strain F2365. Gene expression was quantified by quantitative reverse transcription PCR following growth in nutrient-rich BHI, glutamine-supplemented, or ammonium-supplemented media. (**A**) Expression of genes involved in glutamate synthase, nitrogen assimilation, and glutamine transporter (*gltB*, *gltD*, *gdhA*, *glnR*, and *gluT*). (**B**) Expression of genes associated with the ammonium transport system (*amtB* and *glnK*) and ethanolamine utilization regulators (*eutC* and *eutL*). (**C**) Expression of TCA cycle genes (*citZ*, *citB*, and *citC*). Expression levels are presented as fold change relative to the wild-type strain under the corresponding growth condition. Data represent the mean ± SEM from three replicates. Statistical significance was determined by Student’s *t*-test comparing Δ*gltC* with the wild-type strain. Asterisks indicate statistically significant differences, whereas ns indicates no significant difference.

Because GltC is predicted to regulate components of the glutamate–glutamine metabolic axis, we examined transcriptional responses of key nitrogen metabolism genes under nutrient-rich and defined nitrogen conditions to disentangle growth-independent regulatory effects from nutrient-specific transcriptional responses. Across all tested conditions, GlnR-regulated genes (*gdhA* and *glnR*) and the glutamine transporter gene (*gluT*) were significantly upregulated in the Δ*gltC* strain compared to the wild-type strain F2365 (Fig. 3A). In contrast, glutamate synthase components (*gltB* and *gltD*) were significantly upregulated in BHI and ammonium-rich conditions but significantly downregulated in glutamine-rich medium in the Δ*gltC* strain relative to the wild-type strain, indicating that *gltB* and *gltD* expression is regulated in a condition-dependent manner in the absence of *gltC*. Collectively, these results suggest that loss of *gltC* leads to condition-dependent transcriptional remodeling of nitrogen metabolic pathways.

To determine whether deletion of *gltC* affects utilization of alternative nitrogen sources beyond glutamine, we examined the expression of genes associated with ammonium and ethanolamine metabolism under nutrient-rich and defined nitrogen conditions. Genes associated with the ammonium transport system (*amtB* and *glnK*) and ethanolamine utilization regulators (*eutC* and *eutL*) were significantly upregulated in the Δ*gltC* strain compared to the wild-type strain F2365 under all tested conditions (Fig. 3B). These transcriptional changes suggest a role of GltC in regulating pathways involved in ammonium uptake and ethanolamine utilization.

Because nitrogen metabolism is linked to central carbon metabolism, we next assessed the expression of citrate-associated TCA cycle genes under nutrient-rich and nitrogen-defined conditions. In BHI (Fig. 3C), *citZ* expression was significantly downregulated in the Δ*gltC* strain, whereas *citB* and *citC* showed non-significant increases. In glutamine-rich medium, *citZ*, *citB,* and *citC* were upregulated, although these changes were not statistically significant. In contrast, under ammonium-rich conditions, *citZ*, *citB*, and *citC* genes were significantly upregulated in the Δ*gltC* mutant relative to the wild-type strain. These findings suggest that GltC contributes to the condition-dependent regulation of citrate-associated TCA cycle genes, with the most pronounced effects observed during ammonium utilization.

### Deletion of *gltC* enhances oxidative stress tolerance and activates stress defense and DNA repair pathways

**Fig 4:**
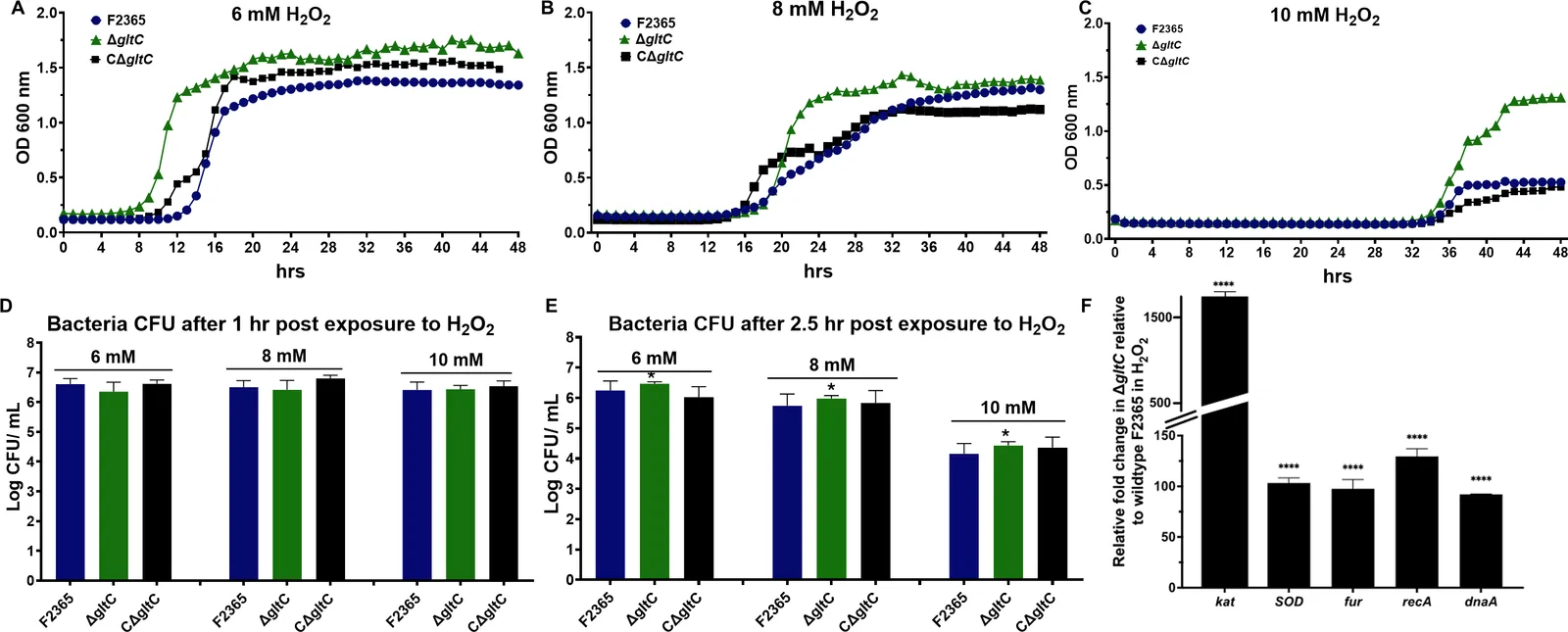
Deletion of *gltC* enhances oxidative stress tolerance and activates stress defense pathways in *L. monocytogenes*. Growth curves in BHI supplemented with 6 mM (**A**), 8 mM (**B**), or 10 mM (**C**) H_2_O_2_. The Δ*gltC* strain exhibited a shorter lag phase than the wild-type F2365 strain when grown in BHI broth supplemented with 6 mM H_2_O_2_. Growth assays were performed in a 48-well plate and repeated at least six independent times with five replicates. Error bars represent SEM. Survival of Δ*gltC* and wild-type F2365 strains after exposure to 6, 8, or 10 mM of H_2_O_2_ for 1 h (**D**) and 2.5 h (**E**). *L. monocytogenes* strains were grown to mid-log phase, resuspended in BHI, and treated with H_2_O_2_, and surviving bacteria were quantified by CFU enumeration on BHI agar plates. (**F**) Relative expression of oxidative stress response and DNA repair genes (*kat*, *sod*, *fur*, *recA*, and *dnaA*) in the Δ*gltC* strain compared with the wild-type strain F2365 following H_2_O_2_ exposure in BHI broth. Gene expression was normalized to 16S rRNA and quantified using the 2⁻^ΔΔCT^ method. Statistical significance was determined using Student’s *t*-test to compare Δ*gltC* with the wild-type strain. Significant differences are indicated by asterisks.

To evaluate the impact of *gltC* deletion on oxidative stress tolerance, growth and survival of the Δ*gltC* and wild-type strains were examined in BHI supplemented with H_₂_O_₂_ at concentrations of 6, 8, and 10 mM. Prior to oxidative challenge, both exhibited comparable growth in BHI, indicating similar baseline fitness under non-stress conditions (Fig. 2A). At 6 mM H_₂_O_₂_, the Δ*gltC* mutant exhibited a markedly shorter lag phase (~3 h) relative to the wild-type strain, indicating faster adaptation to oxidative stress (Fig. 4A). Under moderate oxidative stress (8 mM H_₂_O_₂_), the Δ*gltC* strain displayed consistently higher growth throughout the experiment compared to F2365 (*P* < 0.05) (Fig. 4B), although both strains ultimately reached similar maximal optical densities after 48 h. Under severe oxidative stress (10 mM H_₂_O_₂_), the Δ*gltC* strain exhibited a pronounced growth advantage, with a ~6-h reduction in lag phase and significantly higher growth relative to the wild-type strain (*P* < 0.05) (Fig. 4C).

Survival of the wild-type, the Δ*gltC* mutant, and the complemented strain was further assessed following exposure to H_₂_O_₂_ in BHI for 1 or 2.5 h. After 1 h of exposure to 6, 8, and 10 mM H_₂_O_₂_, survival of the Δ*gltC* mutant was not significantly different from that of the wild-type strain (*P* > 0.05) (Fig. 4D). In contrast, prolonged exposure for 2.5 h resulted in significantly increased survival of the Δ*gltC* mutant relative to the wild-type strain, with differences of approximately 0.4–0.5 log_10_ CFU at 6, 8, and 10 mM H_₂_O_₂_ (Fig. 4E). Collectively, these data indicate that deletion of *gltC* enhances adaptation to oxidative stress, particularly during prolonged exposure to H_₂_O_₂_.

To further investigate the mechanism underlying the enhanced oxidative stress tolerance in the Δ*gltC* strain, we examined the transcriptional responses of key genes involved in oxidative stress defense and cellular repair following H_₂_O_₂_ exposure for 2.5 h. All examined genes were significantly upregulated in the Δ*gltC* strain compared to the wild type following oxidative stress exposure (Fig. 4F), including catalase (*kat*), superoxide dismutase (*sod*), the ferric uptake regulator family member (*fur*), the DNA recombination protein (*recA*), and the replication and repair regulator (*dnaA*). These transcriptional changes indicate an enhanced activation of oxidative stress defense and DNA repair pathways in the absence of *gltC*, consistent with the increased tolerance of the Δ*gltC* mutant to oxidative stress observed in growth and survival assays.

### The expression of cyclic di-adenosine monophosphate regulatory genes is differentially impacted by *gltC* deletion

**Fig 5:**
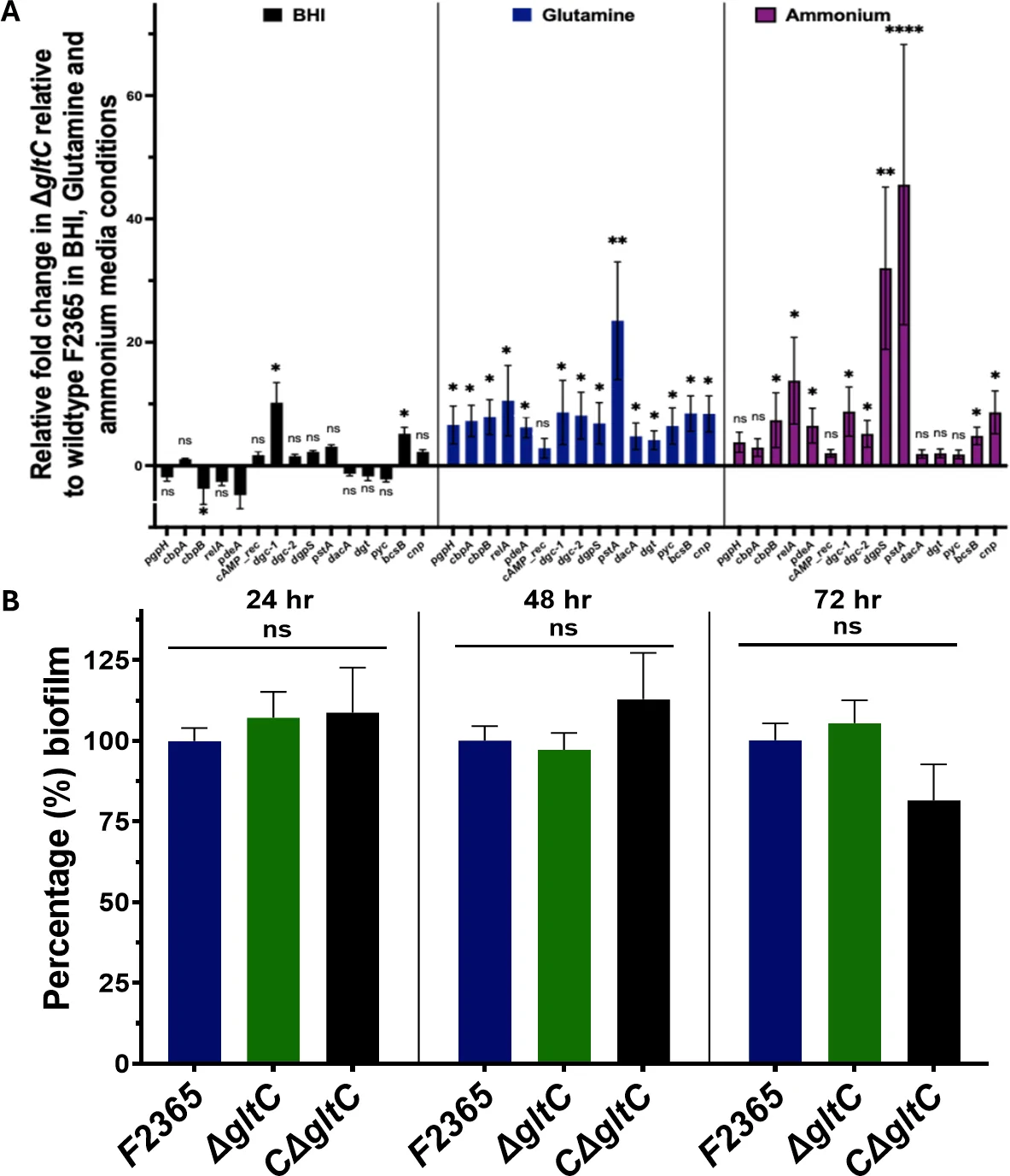
Effect of *gltC* deletion on second-messenger signaling pathways and biofilm formation in *L. monocytogenes*. (**A**) Relative expression of genes involved in c-di-AMP, (P)ppGpp, and cyclic di-GMP metabolism in the Δ*gltC* compared to wild type following growth in BHI, glutamine-rich, and ammonium-rich media. Gene expression was quantified by quantitative reverse transcription PCR (RT-qPCR), normalized to 16S rRNA, calculated using the 2^⁻ΔΔCT^ method, and presented as fold change relative to the wild type. (**B**) Biofilm formation by the Δ*gltC* strain relative to the wild-type strain F2365 (set to 100%) at 24, 48, and 72 h. Biofilm biomass was quantified by crystal violet staining and measured as absorbance at OD_538_ following incubation on polystyrene plates. Data represent the mean ± SEM of three independent biological replicates for RT-qPCR and six biological replicates for the biofilm assay. Statistical significance was determined using Student’s *t*-test comparing Δ*gltC* with the wild-type strain. *P* < 0.05 was considered statistically significant; ns, not significant.

Since GltC contains a binding region for the second messengers c-di-AMP and (p)ppGpp, we investigated the impact of *gltC* deletion on the transcriptional regulation of genes involved in the c-di-AMP, (p)ppGpp, and cyclic di-GMP metabolism (Fig. 5A). Under BHI conditions, the Δ*gltC* strain exhibited significant downregulation of the cyclic di-AMP-binding protein CbpB (*cbpB*) compared with the wild-type strain, whereas the genes encoding the cellulose biosynthesis cyclic di-GMP-binding regulatory protein BcsB (*bcsB*) and the diguanylate cyclase Dgc2 (*dgc2*) were significantly upregulated. Transcript levels of other genes were not significantly altered. In contrast, during growth in ammonium- or glutamine-rich media, deletion of *gltC* resulted in widespread upregulation of the examined genes relative to the wild-type strain F2365, with several genes showing statistically significant increases and others exhibiting consistent but non-significant upward trends. These findings suggest that nitrogen availability strongly influences GltC-dependent regulation of second-messenger signaling networks.

Because c-di-GMP signaling has been implicated in regulating biofilm formation, we next evaluated the effect of *gltC* deletion on biofilm formation under static conditions. Relative to the wild-type strain F2365, the Δ*gltC* strain exhibited a modest increase in biofilm formation of approximately 10% at 24 h and 8% at 72 h, whereas a slight reduction (~3%) was observed at 48 h (Fig. 5B).

### Deletion of *gltC* alters PrfA regulon activity and virulence-associated phenotypes in *L. monocytogenes*

**Fig 6:**
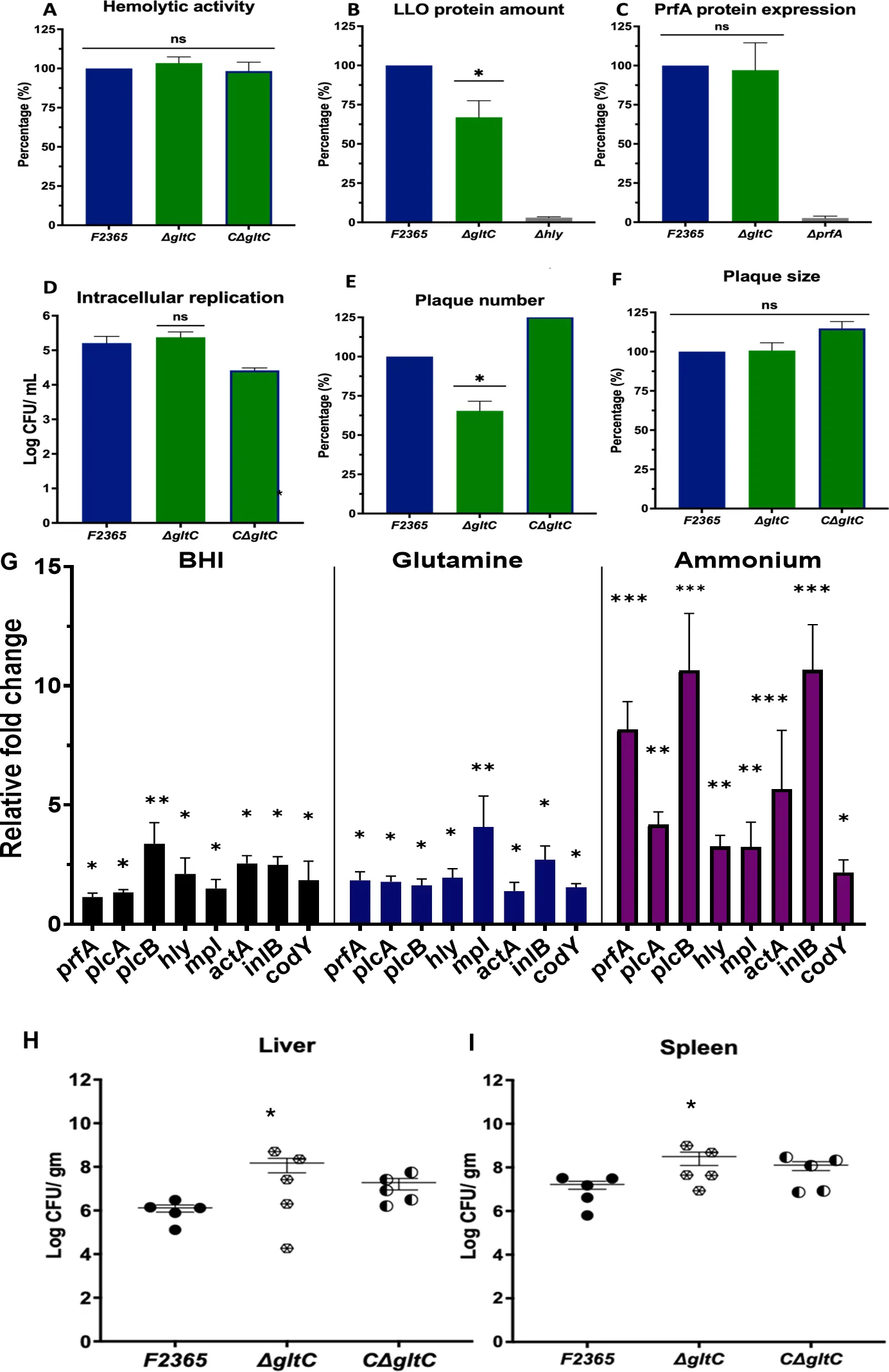
Impact of *gltC* deletion on PrfA-regulated functions, gene expression, and virulence. (**A**) Hemolytic activity of culture supernatants from wild-type F2365 and the Δ*gltC* strain, measured at OD_450_ using a SpectraMax M5 plate reader. (**B**) Western blot analysis of total cellular LLO protein expression, showing a significant decrease in the Δ*gltC* strain compared with F2365. (**C**) Western blot analysis of PrfA protein expression, with no significant difference between the Δ*gltC* and wild-type strains. (**D**) Intracellular replication of Δ*gltC* and wild-type F2365 strains in J774.1 macrophages, determined by CFU enumeration at 5 h post-infection following host cell lysis. (**E**) Plaque diameters produced by the Δ*gltC* strain were not significantly different from those of the wild-type strain. (**F**) Plaque numbers were significantly reduced in the Δ*gltC* strain compared with wild-type F2365. Data in panels A–F represent the mean ± SEM from three independent biological replicates, each performed with at least three technical replicates per condition. Statistical significance was determined using an unpaired two-tailed Student’s *t*-test in GraphPad Prism 9.0. ns, not significant. (**G**) Relative expression of the PrfA regulon and *codY* in the Δ*gltC* strain compared with the wild-type strain F2365 following growth in BHI, glutamine-rich, and ammonium-rich media. Gene expression was determined by quantitative reverse transcription PCR, normalized to 16S rRNA, and calculated using the 2⁻^ΔΔCT^ method. Data represent the mean ± SEM from three independent biological replicates, each performed with technical triplicate. (**H and I**) Bacterial burdens in liver (**H**) and spleen (**I**) of mice infected with the indicated strains. Mice (*n* = 5 per group) were intravenously inoculated with 2 × 10^4^ CFU of the indicated strains, and bacterial burdens were determined 72 h post-infection. Each point represents one mouse. Statistical significance was determined using a nonparametric Mann-Whitney test in GraphPad Prism 9.0. *P < 0.05.

Given that carbon and nitrogen metabolism are tightly linked to regulation of the PrfA virulence regulon (30, 31), we assessed whether deletion of *gltC* alters PrfA-dependent virulence-associated phenotypes. Hemolytic activity, PrfA and listeriolysin O (LLO) protein abundance, intracellular replication, and cell-to-cell spread were evaluated. Hemolytic activity was assessed by measuring sheep erythrocyte lysis mediated by bacterial culture supernatants (32, 33). The Δ*gltC* strain exhibited a non-significant increase (~5%) in hemolytic activity compared with the wild-type strain F2365, whereas hemolytic activity of the complemented strain (CΔ*gltC*) was restored to wild-type levels (Fig. 6A). Western blot analysis of total cellular protein extracts revealed a significant decrease (~40%) in total LLO protein levels in the Δ*gltC* strain, whereas PrfA protein abundance showed a minor, non-significant increase (Fig. 6B and C).

To evaluate whether these PrfA-associated phenotypes impacted intracellular behavior, we assessed intracellular replication and cell-to-cell spread using J774 murine macrophages and L2 fibroblasts, respectively (34, 35). At 5 h post-infection, CFU recovered from infected macrophages revealed an approximately 0.2 log_10_ increase in intracellular bacterial numbers for the Δ*gltC* strain relative to the wild type (Fig. 6D); however, this difference was not statistically significant (*P* > 0.05). Plaque assays revealed no significant differences in plaque diameter among the Δ*gltC*, wild-type, and complemented strains, indicating comparable cell-to-cell spread capacity. However, plaque numbers were significantly reduced (~25%) in the Δ*gltC* mutant compared with the wild-type strain (Fig. 6E and F), suggesting that deletion of *gltC* reduces the efficiency of productive infection events without impairing intracellular spread once infection is established.

To determine whether the altered phenotypes of the Δ*gltC* strain were associated with changes in virulence gene expression, we next examined the transcriptional response of the PrfA regulon under nutrient-rich BHI, glutamine-supplemented, and ammonium-supplemented conditions. Across all conditions, expression of all PrfA regulon genes was significantly upregulated in the Δ*gltC* strain compared with the wild type, including *prfA, plcA, hly, mpl, actA, plcB, inlA,* and *inlB* (Fig. 6G). In addition, expression of *codY*, a global metabolic regulator, was significantly increased in the Δ*gltC* strain relative to the wild type under all tested conditions.

The virulence of the Δ*gltC* strain was evaluated using a murine infection model. At 72 h post-infection, mice infected with the Δ*gltC* mutant exhibited significantly higher bacterial burdens in the spleen and liver compared with those infected with the wild type, with increases of **≈**0.4 and 0.7 log_10_ CFU, respectively (Fig. 6H and I). In contrast, no significant differences in bacterial loads were observed between mice infected with the complemented strain and those infected with the wild type, indicating that genetic complementation restored bacterial burdens to wild-type levels.

## DISCUSSION

GltC is a member of the LTTR family, characterized by an N-terminal DNA-binding region and a central region associated with signal sensing (8). The findings of this study provide a new perspective on the multifaceted role of GltC in the pathogenesis, physiology, nutrient utilization, and stress responses of *L. monocytogenes*, while also clarifying its potential regulatory connection with other virulent determinants.

Importantly, deletion of *gltC* did not affect growth in nutrient-rich BHI or defined media containing glutamine, ammonium, or ethanolamine as sole nitrogen sources, indicating that *gltC* is not required for growth or basic nitrogen utilization under these conditions. These findings are notable given that glutamine assimilation or utilization of other sources of nitrogen such as ammonium and ethanolamine are integral in the growth and physiology of *L. monocytogenes* (12, 19), with glutamine serving as the primary nitrogen source for the synthesis of α-ketoglutarate in the TCA cycle (22, 36). The absence of a growth defect suggests that core nitrogen metabolic pathways remain functionally intact in the absence of *gltC*, likely due to compensatory regulation by other transcriptional regulators. Consistent with the behavior of many LTTRs, the normal growth of the Δ*gltC* strain under both rich and defined nitrogen conditions suggests that GltC is unlikely to function as a constitutive regulator of central metabolism, but instead it may contribute to metabolic reprogramming, stress adaptation, or virulence-associated processes that are not captured by standard growth assays. Furthermore, the lack of a growth defect provides a critical baseline, indicating that any downstream stress- or virulence-associated phenotypes observed in the Δ*gltC* mutant are unlikely to result from general metabolic impairment.

The transcriptional profile of the Δ*gltC* strain indicates that GltC functions as a condition-dependent regulator of nitrogen metabolism rather than acting as a simple on/off controller of glutamate–glutamine biosynthesis. The upregulation of *gdhA*, *glnR*, and *gluT* across all tested conditions suggests that loss of *gltC* induces a nitrogen stress-like response, even in nutrient-rich environments, likely enhancing nitrogen scavenging and assimilation pathways. In contrast, the divergent regulation of *gltB* and *gltD* highlights a role for GltC in integrating glutamine availability signals. Upregulation of these genes in BHI and ammonium-rich media suggests indirect activation under glutamine limitation. However, the downregulation in glutamine-rich conditions indicates that GltC is required to maintain appropriate expression of the GOGAT system when external glutamine is abundant. This pattern suggests that GltC may integrate glutamine availability signals to fine-tune glutamate synthesis, preventing unnecessary energy expenditure while preserving metabolic balance.

The particularly strong transcriptional responses observed under ammonium-rich conditions further suggest that GltC-dependent regulation is influenced by nitrogen availability. Compared with glutamine-rich and nutrient-rich media, ammonium elicited the greatest changes in the expression of genes involved in nitrogen assimilation and central carbon metabolism, suggesting that GltC plays a more prominent regulatory role during ammonium utilization. However, because this study did not directly examine GltC–DNA interactions or intracellular metabolite levels, the molecular basis of this condition-dependent response remains to be determined. Together, GltC appears to contribute to nitrogen homeostasis by coordinating the expression of glutamate synthase, nitrogen assimilation, and transport systems in a nutrient-dependent manner. Importantly, these regulatory effects occur without detectable growth defects, supporting a direct regulatory role rather than a secondary effect of impaired fitness. Together, these findings support a model in which GltC functions as a metabolic regulator that modulates nitrogen utilization strategies in response to nutrient availability, with potential downstream consequences for stress adaptation and virulence.

Another interesting finding of this study is that deletion of *gltC* shifts *L. monocytogenes* toward greater reliance on alternative nitrogen acquisition pathways, as reflected by consistent upregulation of ammonium transport (*amtB* and *glnK*) and ethanolamine utilization (*eutC* and *eutL*) genes across different conditions. This response is consistent with established models in gram-positive bacteria, in which GltC functions as a positive regulator of glutamate synthase, and its loss is sensed as a state of functional nitrogen stress that leads to compensatory activation of GlnR-controlled ammonium uptake and alternative nitrogen sources. Notably, ethanolamine metabolism is relevant in host-associated environments, where ethanolamine is abundant and can serve as both a nitrogen and carbon source (37, 38). Enhanced expression of ethanolamine genes in the absence of *gltC* therefore suggests increased metabolic flexibility that may support survival under nutrient-restricted or host-imposed conditions. Collectively, these findings suggest that GltC may be a negative regulator of the genes responsible for ammonium and ethanolamine utilization in *L. monocytogenes*.

Given the critical role of glutamine metabolism in linking nitrogen assimilation to the TCA cycle, we examined the expression of citrate metabolism genes (*citZ, citB,* and *citC*) to assess GltC’s influence on the nitrogen-carbon balance. Citrate serves dual roles as an essential carbon source for microbial growth and fatty acid biosynthesis at optimal levels, while excess accumulation inhibits gluconeogenic enzymes (39–41) and is associated with defective biofilm formation, increased host immune susceptibility, and impaired virulence (42–44). The deletion of *gltC* in this study causes condition-dependent transcriptional remodeling of citrate metabolism genes, with the most pronounced effect observed under ammonium-rich conditions, where *citZ*, *citB*, and *citC* were all significantly upregulated. This indication suggests enhanced citrate flux into the TCA cycle to sustain α-ketoglutarate pools for glutamate synthesis via the GS-GOGAT pathway when ammonium is the primary nitrogen source. This response may meet elevated energetic or biosynthetic demands associated with alternative nitrogen utilization. Further studies are warranted to quantify citrate levels and evaluate downstream physiological consequences in the Δ*gltC* background.

In this study, the Δ*gltC* mutant exhibited enhanced tolerance to oxidative stress, characterized by significantly shorter lag phases, accelerated growth, and improved survival after exposure to H_₂_O_₂_. This growth advantage was most pronounced under severe stress (10 mM H_₂_O_₂_) and reflects improved stress adaptation rather than acute resistance. Previous studies have shown that deletion of the LysR family transcriptional regulator *oxyR* in *Neisseria gonorrhoeae* resulted in a strain that was highly resistant to H_2_O_2_-induced stress (45, 46), emphasizing the broader role of LysR regulators in modulating oxidative stress responses. At the molecular level, the increased oxidative stress tolerance of the Δ*gltC* mutant was accompanied by significant upregulation of genes involved in oxidative stress defense and DNA repair. Elevated expression of *kat* and *sod* suggests enhanced detoxification of hydrogen peroxide and superoxide radicals (47–49), while upregulation of *recA* and *dnaA* expression reflects activation of DNA repair and replication control mechanisms essential for survival under oxidative damage. Upregulation of *fur* further implies altered iron homeostasis, which may contribute to oxidative stress resistance. Overall, this finding suggests that GltC contributes to the regulation of oxidative stress response pathways and that its deletion promotes enhanced oxidative stress tolerance through coordinated upregulation of oxidative stress defense and DNA repair pathways.

Virulence in *L. monocytogenes* is governed by the PrfA regulon, which coordinates the expression of key determinants required for intracellular survival and systemic infection (25, 50). Our findings suggest that deletion of *gltC* alters PrfA-dependent virulence-associated phenotypes, including a non-significant increase in hemolytic activity, a significant reduction in total cellular LLO protein levels, and significant upregulation of PrfA regulon genes. These results reveal an unexpected link between glutamate-centered metabolism and virulence regulation and suggest that GltC influences PrfA regulon activity, either directly or indirectly. There is limited knowledge about the contributions of *gltC* to hemolysis and LLO expression in *L. monocytogenes*, despite some LTTRs being associated with hemolytic activities in other bacterial species (24, 26, 51, 52).

Biofilm formation is important for the persistence of *L. monocytogenes* on many different surfaces (53). In the present study, deletion of *gltC* did not result in significant differences in biofilm formation, indicating that GltC is unlikely to play a major role in biofilm development under the conditions tested. Despite the paucity of information regarding the contribution of *gltC* to biofilm formation in *L. monocytogenes*, some of the LTTRs (PrhO, BvlR, VtlR, LeuO, and BvlR0) have been found to contribute to biofilm formation in *Ralstonia solanacearum, Pseudomonas aeruginosa, Agrobacterium tumefaciens, Escherichia coli*, *Salmonella enterica*, *Vibrio cholerae*, and *Yersinia enterocolitica* (7, 54–58).

The functional consequences of these transcriptional changes became evident *in vivo*, where mice infected with the Δ*gltC* mutant harbored significantly higher bacterial burdens in the spleen and liver than those infected with the wild-type strain. This finding is consistent with previous reports demonstrating that modest increases in PrfA activity can substantially enhance bacterial survival and dissemination (25, 59, 60). Interestingly, while intracellular replication and plaque size were not significantly altered, plaque numbers were reduced in the Δ*gltC* mutant, suggesting fewer productive infection events despite enhanced virulence once infection is established. Collectively, these findings suggest that GltC contributes to the regulation of PrfA-dependent gene expression and *L. monocytogenes* fitness during systemic infection. This work underscores the central role of metabolic control in coordinating stress adaptation and virulence in *L. monocytogenes*.

In conclusion, this study demonstrates that GltC contributes to coordinating nitrogen availability, carbon metabolism, and stress adaptation in *L. monocytogenes*. Although deletion of *gltC* did not impair growth, it was associated with extensive transcriptional reprogramming of nitrogen assimilation, alternative nitrogen utilization, TCA cycle activity, and second-messenger signaling pathways, reflecting disrupted nitrogen–carbon homeostasis rather than loss of metabolic capacity. Deletion of *gltC* also enhanced oxidative stress tolerance accompanied by upregulation of oxidative stress defense and DNA repair pathways, together with broad upregulation of the PrfA virulence regulon and increased bacterial burdens in the murine spleen and liver. Collectively, our findings support a role for GltC in linking nitrogen metabolism with oxidative stress adaptation and bacterial fitness during systemic infection.

## MATERIALS AND METHODS

### Bacterial strains and growth conditions

*L. monocytogenes* F2365 4b strains and their derivatives were grown in brain heart infusion broth and agar (Difco) at 37°C. *Escherichia coli* DH5α strain was grown in Luria-Bertani (Difco Laboratories) broth and agar (Table 1). Murine macrophage J774A and fibroblast (CRL-2648; ATCC) cell lines were maintained in Dulbecco’s modified Eagle’s medium (DMEM) (ATCC, Manassas, VA, USA) supplemented with 10% fetal bovine serum (Atlanta Biologicals, Norcross, GA, USA) and 1% L-glutamine. Cell cultures were incubated in a humidified atmosphere containing 5% CO_2_ at 37°C and passaged at sub-confluent densities according to the supplier’s recommendations. Antibiotics such as erythromycin (20 µg/mL), ampicillin (50 µg/mL), kanamycin (50 µg/mL), and neomycin (20 µg/mL) were used for selection and maintenance of mutant and complemented *L. monocytogenes* strains, while gentamicin (20 µg/mL) was used for eukaryotic cell culture infection assays to kill extracellular bacteria.

**TABLE 1:** Bacterial strains, plasmids, and primers used in this study^*a*^

Bacterial strain, plasmid, or primer	Description or sequence	Source or reference
Bacterial strains		
*E. coli* DH5α	Competent cells	Standard laboratory strain
*L. monocytogenes*		
F2365	Wild-type serotype 4b strain	(61)
F2365Δ*gltC*	*F2365*Δ*gltC* mutant strain	This study
F2365Δ*gltC::p*PL2-*gltC*	*F2365*Δ*gltC::p*PL2-*gltC* complement strain	This study
Δ*prfA*	Δ*prfA* mutant strain	Portnoy lab
Δ*hly*	Δ*hly* mutant strain	Portnoy lab
Δ*plcA*	Δ*plcA* mutant strain	Portnoy lab
Plasmid		
pHoss1	8,995 bp, pMAD,::*secY* antisense, *ΔbgaB*, Amp^r^, Ery^r^	(62)
*p*PL2	6,123 bp, PSA *attPP,* Chl^r^	(63)
P*gltC* plasmid	pHoss,::Δ*gltC,* Amp^r^, Ery^r^	(62)
*p*PL2-gltC	*p*PL2,::Δ*gltC,* Amp^r^, Ery^r^	This study
Primers used for the construction of Δ*gltC* strain (5′ −3′)		
*gltC-A*	aaaggg**gaattc**CCACGCGCGTATAATAGCAA	EcoRI
*gltC-B*	TTTCCTCCACCTGCAAATGA	
*gltC-C*	TCATTTGCAGGTGGAGGAAAAACAGAACGTATCGGAAGGG	
*gltC-D*	aaaggg**gtcgac**CAAAATCCCCGTACGCCATT	SalI
Primers used for the construction of complemented strain		
*gltC*-CompF1	aaattc**gagctc**GCAGGTGGAGGAAAATAGATG	SacI
*gltC*-CompR1	aaattc**gtcgac**ATTTTGCCATAGCTATTTAACTTGTTC	SalI
Sequence of confirmed deletion		
*gltC*	CCTCTTCAAAAACCGAATCC	
Primers for quantitative reverse transcription PCR (5′ −3′)		
plcA-F	GCATCACTTTCAGGCGTATTAG	
plcA-R	CGTGTCAGTTCTGGGAGTAG	
inlB-F	GATGCGCTTCCTGCTTTAGA	
inlB-R	GAAAGTCCAGCATCCTCCATATT	
recA-F	AGGCGAGCTTGTTGATATGG	
recA-R	CTTCCGTCGATTTCGTACTCTT	
dnaA-F	CCTAGTTACGACACATGGATGAA	
dnaA-R	TCCTGCTCGCCATCAATAAA	
fur-F	CGGTGTTTCTCGGTATGACTT	
fur-R	GTCTGCAATCTGTGCAAATCC	
16S rRNA-F	CAAGCGTTGTCCGGATTTATTG	
16S rRNA-R	GCACTCCAGTCTTCCAGTTT	
LLO-F	CAAATGTGCCGCCAAGAAA	
LLO-R	CGAGAGCACCTGGATATGTTAG	
plcB-F	GCTTGACCGCAAGTGTTCTA	
plcB-R	GATTATCCGCGGACCAACTAAG	
glnR-F glnR-R	AGATCGATGCCGCTGTTT ATTTCGTGCCGGATGGATTA	
gdhA-F gdhA-R	GTTTCCACCCTTCCGTTACT TCAAAGTCAGAGCCACCTTTAC	
eutC-F eutC-R	CAGCGGCAATTGAAGCTAAC TCACCGATTTGGTCCATAGAAG	
eutL-F eutL-R	ATGGCGCTAGCTTTGTTAGT GCTTCACCTTCTGCAACATTC	
amtB-F amtB-R	TCATGGAATCGGAGGTATTTGG GAGAGAAGCATTGCCGTAGAA	
glnK-F glnK-R	AGCACTCGGTACTGGGTTA GGACAGTGGAAACAACGATTTC	
gltD-F gltD-R	GTGATTATCGGTGGTGGAGATAC TCACGCAATTCGGGTAGTTTA	
gltB-F gltB-R	ATGGGCGCAATGGGTTAT CAATCGGAGGGTTGGTTACTT	
plcA-F plcA-R	GCATCACTTTCAGGCGTATTAG CGTGTCAGTTCTGGGAGTAG	
prfA-F prfA-R	ACCAATGGGATCCACAAGAATA GGTAGCCTGTTCGCTAATGA	
citC-F citC-R	TCTGGGACACTTCTTCTTGAAC CTTCTGTCGCACCATCCATTA	
citZ-F citZ-R	GCAACGCTTTCGGATGTTTAT CACCAGCTTGGTCAATTTCTTC	
citB-F citB-R	GAAGGTGGCTATACGACTTACTG CGAGAGGATCCCATTCCATAATC	
mpl-F	GGGAAGAAAGGCTCCGAAATA	
mpl-R	TGCGTAACGCCTAGCTTATC	
actA-F	GAAACAGCACCTTCGCTAGA	
actA-R	CTCTCCCGTTCAACTCTTCTTC	
codY-F	TGAGCGTCTTGGAACACTTATC	
codY-R	GCTACGAGCTTCTTCTTCAATTTC	

^*a*^ Underlined text denotes primer overlap with the gltC-B primer, and boldface in the primer sequences above indicate restriction enzyme sites. Amp^r^, ampicillin resistant; Ery^r^, erythromycin resistant; and Chl^r^, chloramphenicol resistant.

### Construction of *∆gltC* mutant and complement strains

The glutamate synthase activator gene (*gltC*) was deleted in-frame from *L. monocytogenes* F2365 by the allelic exchange technique as previously described (62). Four primers (A, B, C, and D) were designed to amplify the 5′ (A/B) and 3′ (C/D) flanking regions of *gltC* using PCR (Table 1). The upstream and downstream fragments were ligated through overlap extension PCR using A and D primers to generate a deletion construct lacking the *gltC* coding region. The resulting amplicon was cloned into the temperature-sensitive suicide vector pHoss1, propagated in *E. coli* DH5α, and subsequently introduced into F2365 by electroporation to enable chromosomal integration via homologous recombination. Allelic replacement candidates were selected on erythromycin-containing media and screened by PCR to identify clones harboring the in-frame Δ*gltC* allele, which was further confirmed by Sanger sequencing. A complementation strain was constructed by amplifying a DNA fragment containing the entire *gltC* and its promoter from the F2365 genome and ligating it into the pPL2 shuttle integration vector (63). The resulting plasmid was electroporated into *∆gltC* to obtain the complemented strain F2365Δ*gltC*::pPL2-*gltC* (C∆*gltC*), which was verified by PCR and sequencing.

### Growth characterization and nitrogen utilization

The *L. monocytogenes* F2365 4b and Δ*gltC* strains were grown overnight in BHI broth at 37°C with shaking at 180 rpm. Overnight cultures were diluted into fresh BHI and incubated until reaching an optical density at 600 nm of approximately 0.6. A 1-mL aliquot of each culture was harvested by centrifugation (15,000 × *g* for 5 min at 4°C), washed once, and resuspended in phosphate-buffered saline (PBS). To assess the impact of *gltC* deletion on nitrogen source utilization, washed cell suspensions were used to inoculate BHI or MM supplemented with either 20 mM L-glutamine, 5 mM ammonium sulfate [(NH₄)_2_SO_4_], or 30 mM ethanolamine. Cultures were incubated at 37°C with shaking at 180 rpm for 24 h. For kinetic growth measurements, 1-mL aliquots of the inoculated media were dispensed into 48-well plates and incubated at 37°C in a Cytation 5 Cell Imaging Multi-Mode Reader (BioTek) with continuous orbital shaking. OD_600_ was recorded at regular intervals over 24–48 h. All growth and nitrogen utilization experiments were performed in at least three independent biological replicates, each comprising four technical replicates per condition.

### pH assays

*L. monocytogenes* F2365 4b and Δ*gltC* strains were cultured overnight in BHI broth at 37°C with shaking at 180 rpm. When the culture reached an OD_600 nm_ of ~0.6, 1 mL of the culture was harvested by centrifugation and resuspended in PBS. For pH stress assays, BHI broth was adjusted to pH 6.0 using 5 M Tris-HCl and to pH 9.0 using 5 M NaOH. Washed bacterial suspensions were used to inoculate pH-adjusted BHI at a final inoculum of 1%. Growth of wild-type and Δ*gltC* strains at pH 6.0 and 9.0 was monitored in 48-well plates using a Cytation 5 Cell Imaging Multi-Mode Reader (BioTek) at 37°C, with OD_600_ measurements recorded at regular intervals over 48 h.

### Oxidative stress response

The oxidative stress response of Δ*gltC* and C∆*gltC* strains was evaluated in comparison with wild-type F2365 using hydrogen peroxide (H_2_O_2_). Briefly, overnight cultures of bacterial strains were diluted 1:10 in BHI and incubated at 37°C with shaking for ~1 h to reach OD_600_ ~ 0.2–0.3, after which cells were harvested by centrifugation and resuspended in PBS. For survival assays, washed cell suspensions were used to inoculate BHI containing 6, 8, or 10 mM H_₂_O_₂_, followed by incubation at 37°C with shaking. At 1 and 2.5 h post-exposure, 100-µL aliquots were collected, serially diluted in PBS, and plated on BHI agar for enumeration of colony-forming units. In parallel, growth kinetics were monitored by dispensing inoculated H_₂_O_₂_-containing BHI into 48-well plates and measuring OD_600_ at regular intervals over 48 h using a Cytation 5 Cell Imaging Multi-Mode Reader (BioTek) at 37°C with continuous shaking. Oxidative stress assays were performed in three independent experiments, each including six technical replicates per condition.

### Biofilm formation

Biofilm formation by Δ*gltC* and C∆*gltC* was compared to the wild-type F2365 using a static biofilm assay and crystal violet staining, as described previously (64). Briefly, overnight cultures were diluted 100-fold in BHI broth supplemented with 1% glucose (Sigma-Aldrich) and dispensed into 48-well plates, with uninoculated wells included as negative controls. Plates were incubated under static conditions for 24, 48, and 72 h. At the indicated time points, wells were gently washed with PBS, and adherent cells were stained with 0.1% crystal violet (Sigma-Aldrich) for 10 min. Plates were then rinsed with PBS, and the residual crystal violet was solubilized with 70% ethanol. Biofilm formation was quantified by measuring absorbance at 538 nm with a SpectraMax M5 ELISA reader (Molecular Devices, Sunnyvale, CA, USA). Biofilm formation was determined by three independent assays with eight replicates each.

### Hemolytic activity assay

Hemolytic activity of *L. monocytogenes* F2365 and Δ*gltC* was determined as previously described (65). Overnight cultures of bacterial strains were diluted 1:10 in BHI broth and grown at 37°C to reach an OD_600 nm_ of ≈0.7. Bacterial cultures were centrifuged to obtain cell-free supernatants, and 500 µL of each supernatant was mixed with an equal volume of 1% (vol/vol) sheep red blood cell (RBC) suspension prepared in activation buffer, followed by incubation at 37°C for 1 h. After incubation, samples were centrifuged to pellet the intact RBCs, and the clarified supernatants were transferred to 48-well plates for quantification of hemolytic activity by measuring absorbance at 450 nm using a SpectraMax M5 microplate reader (Molecular Devices, Sunnyvale, CA, USA). All assays were performed in three independent experiments, each including three technical replicates.

### Expression of PrfA and listeriolysin O proteins in *L. monocytogenes*

The amount of PrfA and LLO proteins present in Δ*gltC* was compared to F2365 using Western blot with PrfA and LLO polyclonal antibodies (65). Whole-cell protein extracts were prepared as previously described (65). Bacterial pellets were collected by centrifugation, resuspended in cell lysis buffer (50 mN Tris–HCl [pH 8.0], 5% glycerol, 0.5% Triton X-100, 2 nN PNSF, and 1.5 mN EDTA), and incubated at 4°C for 30 min, followed by sonication. DNase I was added, followed by incubation at 4°C for 1 h, and the supernatant was obtained by centrifugation to obtain soluble protein fractions. The proteins were suspended in 4× Laemmli sample buffer containing β-mercaptoethanol, heated for 10 min at 100°C, and resolved by SDS-PAGE, after which they were transferred to PVDF membranes for Western blotting. PrfA was detected using a primary anti-PrfA antibody followed by an HRP-conjugated goat anti-rabbit secondary antibody, whereas LLO expression was detected using anti-listeriolysin O primary antibody with the same secondary antibody. As a loading and expression control, p60 protein was probed using anti-p60 primary antibody and an HRP-conjugated anti-mouse secondary antibody, as previously described (66).

### Intracellular replication

Intracellular replication of Δ*gltC* in macrophages was compared to F2365 using a previously described gentamicin protection assay (34). J774.1 macrophages were grown to confluence, seeded in 48-well tissue culture plates, and infected at a multiplicity of infection of approximately 1–10. After 1 h of incubation to allow bacterial uptake, monolayers were washed with PBS and overlaid with DMEM containing gentamicin (20 µg/mL) to kill extracellular bacteria. After 5-h incubation, cells were washed with PBS, lysed, and intracellular bacteria were released, resuspended, and serially diluted before plating on BHI agar for enumeration. All infections were performed in triplicate and included four technical replicates for each infection.

### Plaque assay

Plaque formation by Δ*gltC* was compared to F2365 using murine L2 fibroblast cells as described previously (35). Fibroblast monolayers were maintained in 75 cm^2^ plastic flasks (Sigma-Aldrich) at 37°C under 5% CO_2_ and seeded at a concentration of 10^6^ cells/well into 6-well tissue culture plates to form a confluent monolayer before inoculation with *L. monocytogenes* strains. After incubation for 1 h, DMEM containing 20 µg/mL gentamicin was added, and plates were incubated at 37°C and 5% CO_2_ for 4 days. Viable cells and plaques were visualized by applying a second overlay consisting of DMEM supplemented with 0.5% agarose and 0.1% neutral red, followed by overnight incubation. Plaque sizes and numbers were determined using a compound microscope with ImageJ, and scores were converted to percentages. This experiment was performed three independent times, each with three technical replicates.

### Gene expression by qRT-PCR

To assess the impact of *gltC* deletion on transcriptional regulation, quantitative reverse transcription PCR was performed to quantify the expression of genes associated with nitrogen sensing and utilization, ammonium and ethanolamine metabolism, TCA cycle activity, oxidative stress defense, second-messenger signaling pathways, and the PrfA-regulon. Target genes and corresponding primer sequences used for qRT-PCR are listed in Table 1. Primer sequences are listed in Table 1 and were designed using Integrated DNA Technologies software. For analysis of nitrogen metabolism-associated genes, overnight cultures were diluted into fresh medium and grown to OD_600_ ≈ 0.6 under nutrient-rich BHI or in MM supplemented with glutamine, ammonium, or ethanolamine. For oxidative stress experiments, exponential-phase cultures were exposed to 8 mM H_₂_O_₂_ for 2.5 h prior to RNA extraction. RNA was extracted using the FastRNA spin kit for microbes and the FastPrep-24 instrument (MP Biomedicals, Santa Ana, CA, USA) using the manufacturer’s instructions. For each strain, total RNA was isolated from three independent biological replicates. Genomic DNA was eliminated from the total RNA by using on-column DNase treatment with an RNase-free DNase set (Qiagen, Hilden, Germany). The quantity and quality of total RNA were analyzed using a NanoDrop ND-1000 spectrophotometer (Thermo Scientific, USA) by measuring the OD_260_/OD_280_ ratio. Purified RNA (5 µg) was used for first-strand cDNA synthesis (Thermo Scientific kit), which was performed with the following steps: 10 min at 25°C, followed by 30 min at 50°C, with reaction termination at 85°C for 5 min. The product of the first-strand cDNA synthesis was diluted 50 times for use in qRT-PCR. qRT-PCR was performed in a 20-µL reaction volume containing 5 µL of cDNA, 10 µL of SYBR Green real-time PCR master mix (Roche Diagnostics GmbH, Mannheim, Germany), 0.6 µL of gene-specific primers (10 µM), and 3.8 µL water. Amplification and detection of specific products were performed using an Mx3000P real-time PCR system (Stratagene) with the following cycle profile: initial denaturation at 95°C for 10 min, 40 cycles of 95°C for 30 s, annealing at 55°C for 30 s, and extension at 72°C for 1 min. Gene expression was normalized to the housekeeping gene 16S rRNA before comparative analysis. Furthermore, DNA melting curve analysis at the end of each run ensured that the desired amplicon was detected and that no secondary products were amplified. For each gene, triplicate assays were performed, and expression levels of tested genes were quantified by the relative quantitative method (2^−ΔΔCT^).

### Assessment of virulence

Swiss Webster mice were obtained from Charles River Laboratories and housed under standard conditions (five mice per cage) with *ad libitum* access to food and water. To assess virulence, the Δ*gltC,* C∆*gltC* strains, and wild-type strain F2365 were diluted in sterile saline to a final concentration of 2 × 10^4^ CFU/mL and administered to mice via intravenous injection through the tail vein (67). Mice injected with sterile saline served as negative controls. At 72 h post-infection, mice were euthanized, and livers and spleens of infected animals were collected, homogenized in saline, and spread on agar plates for CFU determination.

### Statistical analysis

Dot plots and median values of bacterial concentrations for each mouse tissue were generated using GraphPad Prism 9.0. For *in vivo* experiments, a nonparametric Mann-Whitney test was used to assess the statistical significance of bacterial concentrations among the F2365, Δ*gltC*, and C∆*gltC* treatment groups in the liver and spleen of infected mice. Statistical analysis of the hemolytic activity assay, intracellular replication, plaque scores, phospholipase activity, biofilm formation, and oxidative stress was performed to compare Δ*gltC* and wild-type F2365 using Student’s *t*-test. *P* values of <0.05 were considered statistically significant in all analyses. ImageJ was used to quantify bands from Western blot images.

## Data Availability

All relevant data are included in the manuscript.
